# Object Phobia and Altered RhoA Signaling in Amygdala of Mice Lacking RICH2

**DOI:** 10.3389/fnmol.2017.00180

**Published:** 2017-06-08

**Authors:** Tasnuva Sarowar, Stefanie Grabrucker, Tobias M. Boeckers, Andreas M. Grabrucker

**Affiliations:** ^1^WG Molecular Analysis of Synaptopathies, Department of Neurology, Neurocenter of Ulm UniversityUlm, Germany; ^2^Institute for Anatomy and Cell Biology, Ulm UniversityUlm, Germany; ^3^Cellular Neurobiology and Neuro-Nanotechnology Laboratory, Department of Biological Sciences, University of LimerickLimerick, Ireland; ^4^Bernal Institute, University of LimerickLimerick, Ireland

**Keywords:** SHANK3, small GTPase, Rac1, dendritic spine, autism, fear, spine morphogenesis, phobia

## Abstract

RICH2 knockout (RICH2 KO) mice exhibit neophobia in the novel object test. To gain further insight into their anxiety-related phenotype, we subjected these mice to additional behavioral tests to elucidate whether the behavioral abnormality in these mice is a consequence of reduced exploratory motivation, and whether the neophobia is linked specifically to objects or also present for other modalities. RICH2 KO mice engage in normal exploration in a novel environment, suggesting that the anxiety-related phenotype is not due to reduced exploratory drive. Increased fear response was not observed using novel olfactory cues, but restricted to objects. Given that the amygdala is an important brain region mediating anxiety-related behaviors and a prime target for anxiety-related therapeutics, and RICH2 is a Rho-GTPase activating protein (GAP) regulating synaptic spine plasticity via small GTPases, we analyzed spine formation, morphology and receptor composition in amygdala. We found disinhibition of RhoA in the amygdala of RICH2 KO mice, along with a decreased ability for actin polymerization and a reduction in mature spines. However, we detected increased neuronal activation in the amygdala evidenced by c-fos labeling. Thus, we conclude that despite unaltered baseline activity, RICH2 KO mice show heightened amygdala response after exposure to objects, which, however, does not result in homeostatic strengthening of excitatory synapses.

## Introduction

RICH2 (RhoSAP: RhoGAP synapse associated protein) is a protein highly enriched in the post-synaptic density (PSD) of excitatory synapses. It was identified as an interacting partner of SH3 and multiple ankyrin repeat domains 3 (SHANK3) at synaptic spines and to harbor a Rho GTPase activating protein (GAP) domain through which it participates in signaling cascades of small GTPases of the Rho family (cell division cycle 42 (CDC42), Ras -related C3 botulinum toxin substrates 1 (RAC1), and Ras homologous member (RhoA); Raynaud et al., 2013).

Knockout (KO) of RICH2 was previously reported to result in specific behavioral phenotypes including a fear of novel objects, increased stereotypic behavior and impairment of motor learning in mice (Sarowar et al., 2016). In particular, although anxiety in general was not increased in RICH2 KO mice, object neophobia was highly significant. As specific phobias such as irrational fear of objects have been reported in individuals suffering from psychiatric disorders such as Autism Spectrum Disorders (ASD), but rarely in mouse models, RICH2 KO mice are an interesting model to investigate the underlying molecular processes of these behaviors.

The amygdala is a key brain structure involved in processes of fear memory acquisition and storage, but also modulates fear-related learning in other brain structures, such as the cortex and the hippocampus (Ehrlich et al., 2009). In humans, increased activation of the amygdala has been observed in response to angry and contemptuous faces in Generalized Social Phobia (Stein et al., 2002), during emotion processing in anxiety-prone subjects (Stein et al., 2007), and in posttraumatic stress disorder (PTSD; Liberzon and Sripada, 2008). Activation of the amygdala influences cognitive processes, perception, selective attention and explicit memory (Steimer, 2002). Amygdala activation was further related to specific phobias, although the right amygdala seems to play a bigger role in this process (Fredrikson and Furmark, 2003). Additionally, the amygdala may be critical for learning the valence of novel objects from the emotional expressions of others (Blair et al., 2008). In monkeys, it has been shown that lesions of the amygdala disrupted emotional behavior to a set of novel objects (Zola-Morgan et al., 1991). Especially hyperactivity of the basolateral amygdala (BLA) has been associated with excessive/unreasonable fear to a specific object or situation (Forster et al., 2012; Felix-Ortiz et al., 2016).

In rodents, increased activation of the amygdala has been associated with freezing behavior (Holahan and White, 2002) and the processing of aversive stimuli, as well as the consolidation of information that leads to the formation of a specific phobia (File et al., 1998).

However, object neophobia has only been described in few mouse models so far. For example Neuroserpin KO mice show a neophobic response to novel objects (Madani et al., 2003), but the underlying mechanisms are poorly understood.

Here, we specifically investigated the anxiety phenotype of RICH2 KO mice in more detail on behavioral and cellular level. On cellular level, an increase in multi-spine synapses (branched spines) in the hippocampus and cerebellum along with alterations in receptor composition and actin polymerization caused by a disinhibition of synaptic RAC1 was reported in RICH2 KO mice (Sarowar et al., 2016). However, so far, no analysis of amygdala was performed. Thus, here, we investigated whether the observed neophobia is associated with an increased activation of the amygdala and whether an increased activation is caused by synaptic alterations associated with abnormal actin polymerization driven by small GTPases, such as reported before in hippocampus and cerebellum of RICH2 KO mice (Sarowar et al., 2016).

On behavioral level, we investigated whether avoidance of objects in an open field might be caused by a general reduction in locomotor and exploratory activity in RICH2 KO mice. Further, we investigated, whether not only objects, but also other novel stimuli such as odors elicit avoidance behavior and increased anxiety.

## Materials and Methods

### Chemicals and Reagents

For western blots, primary antibodies were purchased from Abcam (RICH2, dilution 1:1000; PSD95, dilution 1:350; CREB, dilution 1:1000; phospho CREB, dilution 1:5000), Novus Biologicals (GAPDH, dilution 1:1000), Santa Cruz (Arp1, dilution 1:1000; EPS8, dilution 1:100), Cytoskeleton (RhoA, dilution 1:500), Millipore (GluN1, dilution 1:500; GluN2B, dilution 1:500; Cortactin, dilution 1:1000), Alomone (GluN2A, dilution 1:400; GluA4, dilution 1:1000), Synaptic Systems (ARC, dilution 1:10,000; c-Fos, diltion 1:1000), Cell Signaling Technology (Histone H3, 1:1000). The secondary antibodies for western blots were purchased from Dako. For Immunohistochemistry (IHC), primary antibodies have been purchased from Abcam (RICH2, dilution 1:500), and Synaptic Systems (Homer1, dilution 1:100; Bassoon, 1:200). For the staining of actin, Alexa488 coupled phalloidin secondary antibody was used from Cytoskeleton with the concentration of 100 nM. SHANK3 antibodies have been described previously for western blot and IHC (Grabrucker et al., 2014). Secondary Alexa-coupled antibodies for IHC were purchased from Invitrogen. Unless otherwise indicated, all other chemicals were obtained from Sigma-Aldrich.

### Animals and Housing Conditions

Generation of the RICH2 KO mouse has been described before (Sarowar et al., 2016). Briefly, KO mice were generated by the insertion of a gene-trap vector causing an additional genomic deletion within RICH2. For behavioral experiments, 70-day old male mice (RICH2^+/+^, RICH2^−/−^, backcrossed for more than 10 generations on C57BL/6J background) were transferred from the animal facility to the behavioral experiment room and habituated for 10 days to the new housing conditions. All animals were housed individually upon arrival in plastic cages under standard laboratory conditions (maintained at 22°C, with lights automatically turned on/off in a 12 h rhythm (lights on at 7 am)) and provided with food and water available *ad libitum*. Heterozygote mice were used for breeding, thus WT and RICH2 KO mice were littermates.

Behavioral experiments were preformed between 9 am and 6 pm. Prior to the behavioral experiments, mice were habituated for 1 h to the test room. All animal experiments were performed in compliance with the guidelines for the welfare of experimental animals issued by the Federal Government of Germany and approved by the Regierungspraesidium Tuebingen and the local ethics committee at Ulm University (ID Number: 0.103 and 1146).

### Behavioral Testing in the Open Field

#### Novel Odor Recognition

The test was conducted in the open field arena (50 × 50 cm). The arena was constructed of white Plexiglas. Mice were first habituated to the open field arena (without any odor inside) for 30 min and placed back into the home-cage for approximately 1–2 min. In the meantime, two drops of water (100 μl) were placed in the arena, approximately 4 cm from the sidewall. The mouse was placed back into the same arena facing the opposite side of the objects and allowed to freely explore the setting for 10 min. After this acquisition period, the mouse was placed back into the home cage. The water drops were replaced with the same amount of banana-flavored water and the mouse was placed in the arena again. The mouse explored the arena freely for 10 min and was recorded. Afterwards, one of the banana-flavored water drops was replaced with an almond-flavored water drop, while the mouse was in the home cage. Then again the mouse was allowed to freely explore the arena and a video was recorded for 10 min.

#### Novel Object Exploration

Like novel odor exploration, each mouse was allowed to explore an open field arena for 30 min as habituation phase. Afterwards, two identical objects were placed in the arena at the same position as the water drops. The mice were allowed to freely explore the objects for 10 min. After this acquisition period, mice were placed back in the home cage. Afterwards, one of the identical objects was replaced with a white playmobil® horse and the mouse was allowed to freely explore for 10 min. Finally, in the last acquisition phase, the horse was replaced with another novel object and the mouse was allowed to freely explore for 10 min.

#### Quantification of Open Field Behavior

Videos were acquired using a CCD camera (Conrad CCD camera S/W). Activity parameters such as distance traveled, time spent in the object zone vs. time spent in the non object zone, zone transition etc., during all of the different test sessions were scored using the video tracking software EthoVision XT (Noldus, Wageningen, Netherlands).

### Isolation of Amygdala from Whole Brain

The amygdala was isolated from the whole brain according to Zapala et al. (2005). Briefly, two coronal cuts were made at approximately Bregma −1 and −2.75. The section was put down with the caudal side facing up and incisions were made in a triangular shape both at the right bottom and left bottom, avoiding hippocampus. These brain tissues contained amygdala and further procedures were carried out according to the desired experimental protocol. For brain sectioning, sections from Bregma −2.8 (approximately) were used for staining and histological procedures.

### Western Blot-Analysis

Western blot experiments were performed using PSD-enriched P2- and nuclear fractions of the amygdala. The amygdala brain regions were isolated from three males WT and three RICH2 KO mice (P70–P80) and were dissolved in HEPES buffer (10 ml buffer/gram tissues; 10 mM HEPES; 0.32 M Sucrose, pH 7.42; Protease Inhibitor Cocktail tablet (Roche)). Tissues were homogenized using sonication to obtain crude homogenate. The crude homogenates were centrifuged at 3200 rpm for 15 min at 4°C, resulting in nuclear fraction (P1) and soluble supernatant (S1). The S1 fractions were further centrifuged at 11,400 rpm for 20 min at 4°C. Subsequently, the pellet or synaptosomal fraction (P2) was dissolved in ice-cold HEPES buffer. The protein concentration was measured using Bradford analysis and 10 μgof protein was loaded on PAGE in 4× SDS sample loading buffer.

### Real-Time PCR

Total RNA was isolated from WT and RICH2 KO mice amygdala using the RNeasy kit (Qiagen) according to the manufacturer’s instructions and protocol. cDNA synthesis using reverse transcriptase and DNA amplification using polymerase were carried out simultaneously via Transcriptor One-Step RT-PCR Kit (Roche, Germany), using RICH2 genotyping primers (WT/KO: 5-AGC TAG CAG ACG CTT CAA GG-3, WT: 5-CCA ACA AAG CTG AAA GCA CA-3, KO: 5-CAC ATC CAT GCT GAG GAT GA-3). The amplified products were visualized using gel electrophoresis.

### Immunohistochemistry (IHC)

#### Fluorescent IHC

Cryosections (thickness 16 μM) were thawed and fixed in paraformaldehyde (PFA) for 20 min in a hydrated box. Then the sections were washed three times in 1× PBS for 5 min, followed by permeabilization with 0.2% triton in 1× PBS for 1 h. Afterwards, the slices were washed again three times with 0.05% triton in 1× PBS, 10 min each time. Later blocking of antigens was done using an incubation with 10% FCS for 2 h. Then, the slices were incubated with primary antibody diluted in blocking solution at 4°C overnight. Subsequently, the slices were washed with 0.05% triton in 1× PBS for 10 min, followed by incubation with secondary antibody diluted in blocking solution at 37°C for 2 h in dark. After washing three times, 15 min each with 1× PBS containing 0.05% triton, the slices were incubated 5 min with PBS containing DAPI, and after a final washing step with ddH_2_O for 5 min, slices were mounted with VectaMount (Vector Laboratories).

#### c-Fos Staining

Thirty minutes after the behavioral experiments, the mice were sacrificed via cervical dislocation. Brains were taken out and snap frozen in liquid nitrogen. Brain sections (30 μM thickness) were prepared and fixed in 4% PFA for 1 h. After washing three times with 1× PBS 5 min each, the sections were permeabilized with 0.2% triton in 1× PBS solution for 45 min. Then, the slices were washed three times with 1× PBS, 5 min each. Afterwards, the slices were incubated in 1% H_2_O_2_ in 1× PBS solution for 20 min. After three washing steps, blocking was done using 2% goat serum in 1× PBS. Next, the slices were incubated with c-Fos antibody (purchased from Synaptic Systems) 1:5000 diluted in blocking solution at room temperature overnight. The following day, the slices were incubated with secondary goat anti rabbit antibody for 1 h after three washing steps (1× PBS). After the incubation, the slices were washed three times again with 1× PBS and later incubated in 3,3′-Diaminobenzidine (DAB) solution (10 mg DAB in 100 ml 1× PBS, with 200 μl NiCl and 30 μl 30% H_2_O_2_). Then, slices were first washed three times in 1× PBS; later washed with 70% (once), 90% (once) and 100% (twice) ethanol, for 5 min each time. Finally, the slices were washed with xylene three times, 5 min each and mounted with Entellan (Merck Millipore). The sections were scanned using a BZ-X700 from Keyence.

### *In Situ* Hybridization

*In situ* hybridization was performed according to protocols described previously (Boeckers et al., 1999; Laube et al., 2002). The brain tissues were first frozen, cryo-sliced with 8 μm of thickness and then frozen on Super Frost Plus slides. The sections were stored at −70°C. Transcripts encoding RICH2 were detected with antisense oligonucleotide purchased from MWG Eurofins (Ebersberg, Germany) directed against the 5′ and 3′ ends of the mRNA: TGA GCT TCT TGT GTG TGC TGT GGG ACA CCT GTT T.

### Golgi Staining

Golgi staining was performed on whole brains from WT and RICH2 KO animals (*n* = 5 for each group, age p70–80) using the FD Rapid GolgiStain Kit (FD NeuroTechnologies) according to the manufacturer’s instructions. In order to obtain the 3D overview of the dendritic spines, *Z* stack images of Golgi stained slices were made with a BZ-X700 from Keyence. For a general overview of the cell, 40× magnification was used and to zoom into individual dendrites, 63× magnification was used. 10–15 stacked images were taken at 40× magnification and 20–25 stacked images were taken at 63× magnification (0.1 μm per z-stack). For the quantification of spine density, the length of the dendrite was measured using the freehand lines tool from ImageJ. Then, the number of spines was documented for the respective length of the dendrite. In total, 429 (WT) and 450 (KO) μm of dendritic length were quantified in this way. The total number of spines for unit length was calculated for each animal and averaged for each group. The spine morphology was quantified based on the length, width and head-to-neck ratio of each distinct spine and the spine assigned to a morphological category. The criteria for each spine morphological category (filopodia, thin, stubby and mushroom spines) were: filopodia (length > 2 μm), thin (length < 1 μm), stubby (length: width ratio approx. 1:1, no spine neck visible), mushroom (width > 0.6 μm; Risher et al., 2014). Spine morphology was assessed from primary dendrites. For better visualization of the 3D view of spines, scrolling through the different stacks was done. In total 101 spines (for WT animals) and 94 spines (for KO animals) were categorized. Each morphological category was quantified as percentage of total spines for each animal and averaged for each group.

### RhoGAP Assay

RhoGAP assays were performed on P2 lysates of brain amygdala from WT and RICH2 KO animals using the RhoGAP Assay (BK 105, Cytoskeleton) according to the manufacturer’s instructions and protocol. The absorbance was measured using a Cytation 3 microplate reader from BioTek. Upon converting the GTP-bound active small GTPases to GDP-bound inactive small GTPases, RhoGAPs liberate phosphate. The generated free phosphate can be quantified using the Cytophos reagent, which is provided with the kit. To evaluate the GAP activity of tissue lysates, a standard curve was generated using different concentrations of KH_2_PO_4_. The tissue lysates from WT and KO amygdala were incubated with GTP and Cytophos. Then, the absorbance was measured and plotted on the standard curve, and thus, the GAP activity of each lysates was quantified. The small GTPases (RhoA, RAC1, CDC42, RAS p21) were provided with the kit. To determine the GAP activity of the lysates on each small GTPase, the corresponding GTPase was incubated with and without lysates (as a control), along with GTP. Later the liberated phosphate was quantified using Cytophos. The absorbance in presence of lysates (both WT and KO) and control state (Ctrl) is presented as bar graph.

### Actin Polymerization Assay

Actin polymerization assays were performed with the P2 amygdala lysates of three WT and three RICH2 KO mice using the Actin Polymerization Biochem kit from Cytoskeleton (Cat. BK 003) according to the manufacturer’s instructions and protocol. The fluorescence was measured using a Cytation 3 microplate reader from BioTek.

### Statistics

Statistically significant differences are indicated in the figures by *p ≤ 0.05, **p ≤ 0.01 and ***p ≤ 0.001. Standard errors in graphs represent SEM. *P* values between 0.05 and 0.1 are mentioned as trend in the manuscript. Obtained values were tested for normal distribution using Q-Q plot from SPSS.

#### Signal Intensities

Fluorescence images were obtained using an upright Axioscope microscope equipped with a Zeiss CCD camera (16 bits; 1280 × 1024 pixels per image) using the AxioVision software (Zeiss) with the same exposure time throughout the experiment and all of the conditions, and were analyzed using ImageJ 1.51a. Statistical analysis was performed using Microsoft Excel for Windows and tested for significance using unpaired *t*-tests. All values were normally distributed.

#### Western Blot Quantification

Images of bands were taken using a MicroChemi 4.2 imaging device (Biostep) with GelCapture version 2.0 software. Western blot bands were quantified using ImageJ. All WB bands were normalized to GAPDH and the ratios averaged and tested for significance using unpaired *t*-tests.

#### Behavior

WT controls and RICH2 KO littermates were compared for each behavioral task using unpaired *t*-test. Statistical analysis was preformed with SPSS version 20.

## Results

### RICH2 is Expressed in Amygdala Neurons

**Figure 1:**
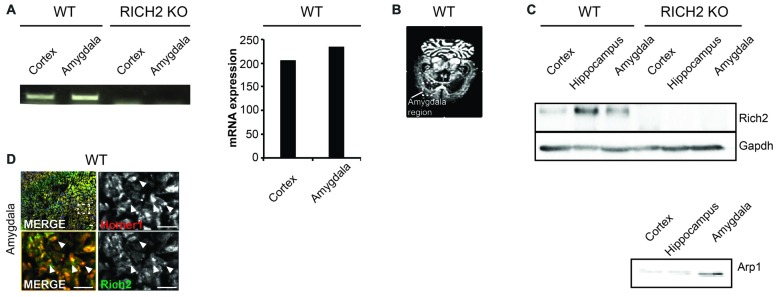
RICH2 is expressed at glutamatergic synapses in the amygdala. **(A)** Expression of *Rich2* was detected on mRNA level using a PCR approach in amygdala using lysate from wild type (WT) mice. Cortex lysate was used as control as expression of *Rich2* has been reported in cortex previously. The expression levels of *Rich2* in amygdala were slightly higher compared to cortex. The primers used for amplification of *Rich2* were placed in a region deleted in the genome of RICH2 Knockout (KO) mice. Absence of *Rich2* expression in amygdala of RICH2 KO mice was confirmed. **(B)** *In situ* hybridization confirms *Rich2* expression in the amygdala in adult WT animals. **(C)** RICH2 protein was not detected in RICH2 KO mice, but in amygdala of WT mice, and hippocampus and cortex as reported previously. ARP1 is a protein enriched in amygdala and was used as control to ensure proper preparation of amygdala tissue. **(D)** Immunocytochemistry performed on brain sections of WT mice reveals RICH2 signals co-localizing with HOMER1, a marker for excitatory postsynapses, in the amygdala. Top left: overview indicating magnified area in bottom left and right images. Arrows indicate clearly co-localizing signals (scale bar = 20 μm).

RICH2 KO mice used in this study have been characterized before and a *Rich2* specific genomic knock-out confirmed by several experimental approaches (Sarowar et al., 2016). RICH2 is a protein predominately expressed in the brain (Sarowar et al., 2016). Within the brain, expression analysis reveals the presence of *Rich2* mRNA in amygdala (Figure 1A). *In situ* hybridization using adult WT brain further confirms expression of *Rich2* in the amygdala (Figure 1B). Further, RICH2 was detected on protein level through analysis of lysates of several brain regions by Western blot. RICH2 expression was found in cortex and hippocampus as reported before, but also in lysate from amygdala region (Figure 1C). To control successful isolation of amygdala, detection of Arp1 as marker protein (Zirlinger et al., 2001) was used (Figure 1C). RICH2 was found mainly co-localizing with HOMER1, a marker for glutamatergic excitatory post-synapses in the amygdala (Figure 1D). Thus, given the presence of RICH2 in amygdala, altered amygdala circuitry through abnormal RICH2 signaling at synaptic spines may contribute to the increased behavioral inhibition in response to novel objects as observed previously.

### Specific Object Neophobia in RICH2 KO Mice

RICH2 KO mice were shown to display significantly decreased object approaches in a novel object test before (Sarowar et al., 2016). Given that this behavioral inhibition may be the consequence of a general reduction in the motivation to explore, we tested this hypothesis by examining levels of exploratory activity during the habituation phase of two test paradigms, an olfactory test and a novel object test.

**Figure 2:**
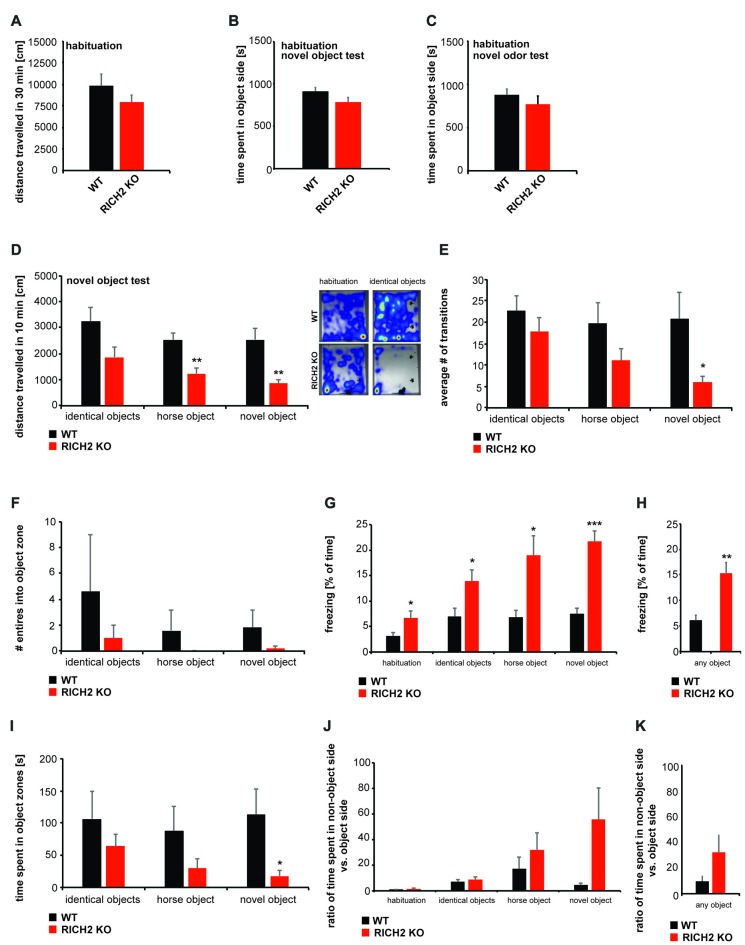
Normal motivation to explore but object neophobia in RICH2 KO mice. **(A–K)** Habituation to the test arena for the novel object test and exposure to novel odors was evaluated in WT and RICH2 KO mice. Habituation was performed for 30 min. **(A)** No significant difference in the distance traveled was detected between RICH2 KO and WT mice in the habituation phase in the open field used for odor exposure. **(B)** No innate side preference was detected for the test arena of the novel object test and odor test, and no difference in the time spent in the designated objet **(B)** or odor **(C)** zone was found (unpaired *t*-test, *p* = 0.1034 for **(B)**, *p* = 0.3718 for **(C)**, *n* = 5 for each group). **(D)** During the 10 min of exposure to objects (identical objects, one novel object, and horse shaped object), RICH2 mice showed differences in the distance traveled compared to WT mice. A decrease was seen in response to novel objects (as trend) and a significant decrease in response to horse and novel object (unpaired *t*-test, *p* = 0.0703 for identical object, *p* = 0.0062 for horse, and *p* = 0.0065 for novel object, *n* = 5 in each group). **(E)** The number of transitions into the object side was decreased in RICH2 KO mice (significant for the novel object; unpaired *t*-test, *p* = 0.0481, *n* = 5 for each group). **(F)** The number of entries into the object zone were decreased in RICH2 KO mice (unpaired *t*-test, *p* = 0.4437 for identical object, *n* = 5 for each group). **(G)** Decreased locomotion was accompanied by a significant increase in freezing behavior seen in RICH2 KO mice (unpaired *t*-test, *p* = 0.0368 (habituation); *p* = 0.0376 (identical object); *p* = 0.0170 (horse); *p* = 0.0003 (novel object), *n* = 5 in each group). **(H)** Averaging freezing behavior across all experiments with objects present, a significant increase in RICH2 KO mice compared to WT is visible (unpaired *t*-test, *p* = 0.0046, *n* = 5 for each group). **(I)** The time spent in object zones was decreased for horse object and novel object (unpaired *t*-test, *p* = 0.3882 (identical objects); *p* = 0.1924 (horse)), and significantly decreased for the novel object (*p* = 0.0458; *n* = 5 in each group). **(J)** Comparing the time spent in the non-object side of the arena with the object side, RICH2 KO mice spent more time in the non-object side compared to WT mice (unpaired *t*-test, *p* = 0.2662 (habituation); *p* = 0.5067 (identical object); *p* = 0.3728 (horse); *p* = 0.0735 (novel object), *n* = 5 in each group). **(K)** Averaging the ratios between the time spent in non-object zone and time spent in object zone, an increase (seen as trend, *p* = 0.09) in time spent in the non-object is confirmed in RICH2 KO mice.

We detected a slight but not significant decrease in locomotor behavior in RICH2 KO mice during the habituation phase for the novel object and novel odor test (Figure 2A). Thus, except from a slight reduction in locomotion, possibly due to increased anxiety/freezing in RICH2 KO mouse, exploratory behavior in the habituation phase was not different between WT and RICH2 KO mice. Reduced approach behavior in subsequent tests therefore cannot be explained by reduced exploratory activity in RICH2 KO mice.

To investigate the response of RICH2 KO mice compared to WT mice elicited by the presence of objects, and novel objects with specific shape, we measured exploration in a familiar environment using the novel object exploration test (Figures 2B–K). During the habituation phase, no innate side preference was detected in RICH2 KO or WT mice in both test paradigms, the object test and test with exposure to novel odors (Figures 2B,C). After habituation, two identical objects were placed in an open field. RICH2 KO mice showed a decrease in the distance traveled in response to the presence of the objects compared to WT mice (Figure 2D). Replacement of a familiar object with a novel object—either in horse shape or alternative inorganic shape as the first object, similarly elicited a significant reduction in the distance traveled of RICH2 KO mice (Figure 2D). There was no significant difference observed in the response regarding the type and shape of the objects (one-way ANOVA: WT *F*_(2,12)_ = 3.212; *p* = 0.076; RICH2 KO *F*_(2,12)_ = 3.322; *p* = 0.071).

The number of transitions was reduced as a trend in presence of the identical objects and novel horse objects, and significantly in presence of the novel object (Figure 2E). The number of entries into the zone around the object (object zone) was decreased in RICH2 KO mice or almost absent (horse object; Figure 2F). There was no significant difference observed in the response regarding the type and shape of the objects (one-way ANOVA: WT *F*_(2,12)_ = 0.361; *p* = 0.704; RICH2 KO *F*_(2,12)_ = 0.808; *p* = 0.469).

The decreased locomotion and entries into object zones was accompanied by a significant increase in freezing in RICH2 KO mice (Figures 2G,H). The time spent in object zones was decreased (identical objects and horse object, and significantly for novel object) in RICH2 KO mice compared to WT (Figure 2I). Compared to the time spent in the object side of the arena, RICH2 KO mice spend more time in the non-object side as WT mice (horse object and novel object, seen as trend; Figures 2J,K). There was no significant difference observed in the response regarding the type and shape of the objects (Figure 2G: one-way ANOVA: WT *F*_(3,16)_ = 0.277; *p* = 0.841; RICH2 KO *F*_(3,16)_ = 0.159; *p* = 0.922, Figure 2I: WT *F*_(2,12)_ = 0.236; *p* = 0.793; RICH2 KO *F*_(3,16)_ = 1.245; *p* = 0.323; Figure 2J: WT *F*_(3,16)_ = 2.218; *p* = 0.126; RICH2 KO *F*_(3,16)_ = 2.428; *p* = 0.116). Thus, the results confirm increased anxiety in response to objects in RICH2 mice.

**Figure 3:**
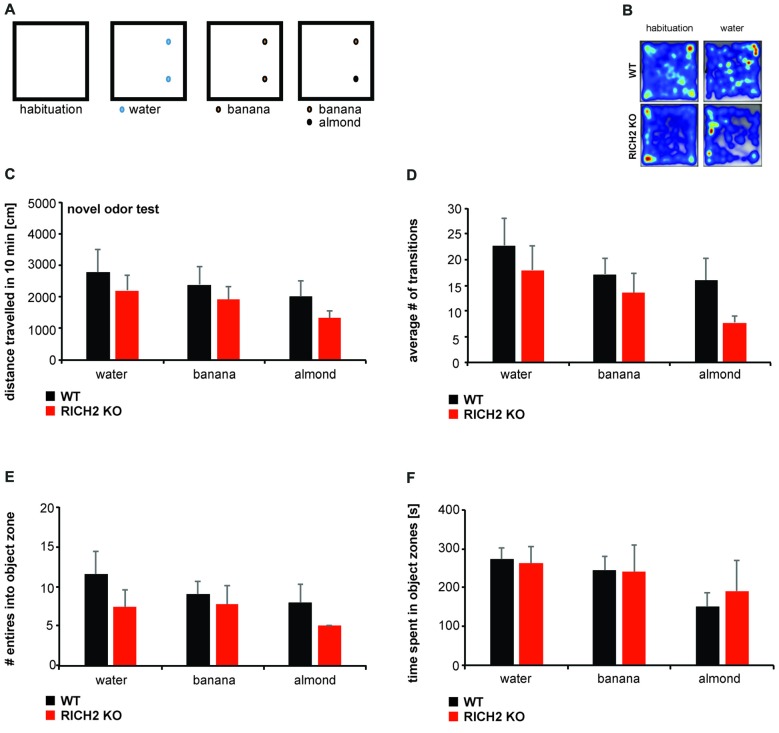
RICH2 KO mice display no neophobia in response to odors. **(A)** Mice were exposed to water (control), banana, and almond (test) odors for 10 min. Odors were introduced in similar spots as objects in the open field and the response of mice assessed **(B)**. **(C)** No significant difference in the distance traveled (*p* = 0.5308 for water, *p* = 0.5045 for banana, *p* = 0.2682 for almond, unpaired *t*-test), the number of transitions **(D)** (*p* = 0.5078 for water, *p* = 0.4687 for banana, *p* = 0.1036 for almond, unpaired *t*-test), the entries into object zones **(E)** (*p* = 0.2797 for water, *p* = 0.6869 for banana, *p* = 0.2288 for almond, unpaired *t*-test) and time spent in object zones **(F)** (*p* = 0.8350 for water, *p* = 0.9398 for banana, *p* = 0.6450 for almond, unpaired *t* test) was detected between RICH2 KO mice and WT in response to novel odors (*n* = 5 for each group).

Next, to further assess whether the observed increase in behavioral inhibition is caused only by objects, or also other novel stimuli, we exposed RICH2 KO mice to several olfactory cues that are normally not aversive for mice (Figures 3A,B). WT and RICH2 KO mice were exposed to water (control), banana, and almond (test) odors for 10 min. No significant difference for water, banana and almond in the distance traveled (Figure 3C), the number of transitions (Figure 3D), the entries into object zones (Figure 3E), and time spent in object zones (Figure 3F) was detected between RICH2 KO mice and WT in response to novel odors. Thus, anxiety in RICH2 mice is triggered by objects but not by olfactory cues.

### Altered Activation of Amygdala Neurons in RICH2 KO Mice in Response to Novel Objects

**Figure 4:**
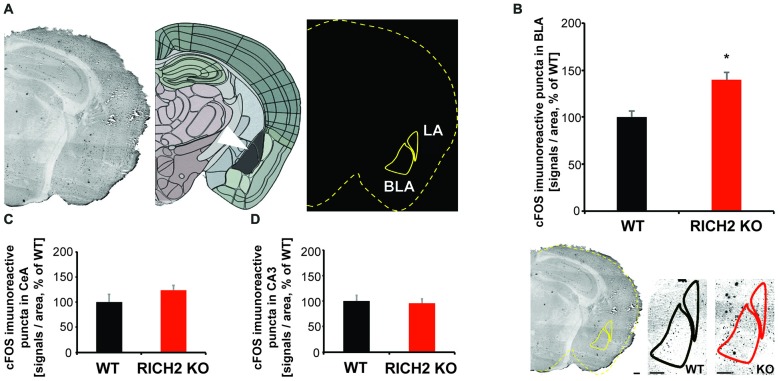
Altered neuronal activation in amygdala of RICH2 KO mice in response to novel objects. **(A)** c-FOS staining was performed 30 min after the novel object test. An overlay mask was made outlining the brain section and location of basolateral amygdala (BLA) and CeA using the mouse brain atlas at approximately Bregma −2.8. The mask was merged with the scanned slices and the number of immunoreactive signals within the BLA and CeA region was counted. **(B)** The number of c-FOS stained cells is significantly increased in the amygdala (BLA) of RICH2 KO mice (Unpaired *t*-test, *p* = 0.0169, *n* = 3 per group). **(C)** A non-significant increase was detected in the CeA. **(D)** As control, c-FOS staining was evaluated in the hippocampal CA3 region. No significant difference between WT and RICH2 KO mice was detected. CeA, central amygdala; LA, lateral amygdala; BLA, the lateral (LA) and basal (BA) nuclei (together referred to as the basolateral amygdala—BLA). Scale bar = 300 μm.

To assess whether the amygdala plays a role in this behavioral abnormality of RICH2 KO mice, we measured the activation state of amygdala neurons 30 min after the novel object test by quantification of c-fos staining (Figure 4A). Expression of c-fos is an indirect marker of neuronal activity, as c-fos expression is increased in active neurons (Cruz et al., 2013), and it has been reported that an increase of cFos expression occurrs 30 min after neuronal activation (Szyndler et al., 2009; Zhong et al., 2014). We have focused on the BLA (the lateral and basal nuclei together), since it was shown that lesions in the BLA are able to attenuate taste neophobia (Lin et al., 2009; Gómez-Chacón et al., 2012), and diminish neophobic responses (Nachman and Ashe, 1974), especially in presence of additional hippocampal pathology (Aggleton et al., 1989). The BLA may modulate memory consolidation in recently active synapses in efferent brain regions (McReynolds et al., 2014), and food presented in a novel way, elicits avoidance in rats that is positively correlated with the percentage of BLA area (Kiyokawa et al., 2017).

Our results show that the expression of c-fos is significantly increased in the BLA of RICH2 KO mice compared to WT (Figure 4B). Thus, it is possible that RICH2 KO mice suffer from a hyper-activation of amygdala neurons. This increased activation may be caused by altered input into amygdala and/or altered post-synaptic plasticity and synapse maturation in signal receiving neurons in the amygdala. In the central amygdala (CeA) c-fos expression was also slightly increased in RICH2 KO mice (Figure 4C). As control, the hippocampal CA3 region was measured and no significant differences in the expression of c-fos were detected (Figure 4D).

### Altered Synaptic RhoA Signaling in the Amygdala of RICH2 KO Mice

**Figure 5:**
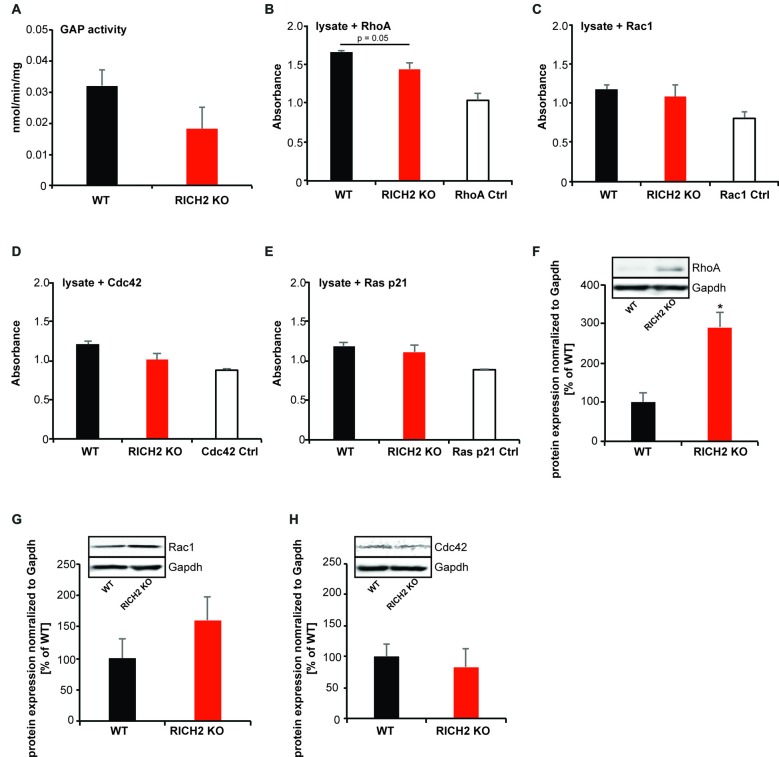
Altered Rho GTPase signaling in amygdala of RICH2 KO mice. **(A)** Comparison between the GTPase activating protein (GAP) activity of the P2 lysates from WT and RICH2 KO mice. The GAP activity is measured in nmoles/min/mg of small G-protein. First a standard curve is made using the absorbance of KH_2_PO_4_ at different concentrations, later the absorbance of WT and RICH2 KO lysates are plotted on the graph. The GAP activity is higher for WT lysates compared to RICH2 KO lysates. **(B)** In the presence of the small G protein RhoA, the absorbance of WT lysates is higher than that of RICH2 KO lysate, which indicates that RICH2 is acting as a GAP for RhoA in amygdala. **(C)** There is no difference between the absorbance of WT and RICH2 KO lysates in the presence of RAC1 and CDC42 **(D,E)** Like RAC1 and CDC42, RAS p21 is not a target G protein for RICH2 in amygdala as there is no difference in the absorbance of WT and RICH2 KO lysates in the presence of RAS p21. **(F)** Western blot analysis of amygdala P2 lysates reveals that expression of RhoA is significantly higher in RICH2 KO lysates, in comparison with WT lysates (Unpaired *t*-test, *p* = 0.0508, *n* = 3). **(G,H)** Western blot analysis revealed no alteration in the expressions of Rac1 **(G)** and Cdc42 **(H)** between WT and RICH2 KO using amygdala P2 lysates.

Thus, next, to investigate the underlying mechanisms causing abnormal activation of amygdala neurons, we measured the activity of small GTPases. RICH2 has RhoGAP activity and thus is able to promote GTP hydrolysis and thereby inactivate Rho GTPases such as RhoA, RAC1 and CDC42 (Raynaud et al., 2014). Previously, we have shown that loss of RICH2 leads to disinhibition of RAC1 in the hippocampus, thereby promoting spine growth and the formation of multiple spine synapses via increased actin polymerization (Sarowar et al., 2016). However, *in vitro* studies, activity of RICH2 was also reported for RhoA (Raynaud et al., 2014). Thus, we analyzed whether effects of RICH2 KO on Rho GTPases can be detected in amygdala lysate. In this assay, the small G-protein is incubated in presence of GAP (i.e., RICH2 in amygdala lysate) and GTP. Subsequently, GTP hydrolysis is quantified via CytoPhos reagent by measurement of the amount of phosphate generated through determination of absorbance. A reduction in hydrolysis using lysate of RICH2 KO mice compared to WT in presence of RhoA, RAC1, CDC42 or RAS p51 would indicate activity of RICH2 for the corresponding small GTPase protein. The results show that the GAP activity is higher for WT lysates compared to RICH2 KO lysates, suggesting that the deletion of RICH2 has reduced the GAP activity (Figure 5A).

To investigate which Rho GTPase family members is affected most by RICH2 KO in the amygdala, we added the small G protein RhoA, RAC1, CDC42 and RAS p21 to the lysate (Figures 5B–E). In the presence of RhoA, the absorbance of WT lysates is higher than that of RICH2 KO lysate, which indicates that RICH2 is acting as a GAP for RhoA in amygdala (Figure 5B). There was no difference between WT and RICH2 KO lysates in the presence of RAC1 and CDC42 suggesting that RAC1 and CDC42 are not target G proteins for RICH2 in amygdala (Figures 5C,D). Similarly, no difference between WT and RICH2 KO lysates was found in the presence of RAS p21 (Figure 5E). Deletion of RICH2 leads to a significant increase of RhoA protein at synapses in the amygdala (Figure 5F) that may be specific to synapses or reflecting a general increase of RhoA levels. The expression levels of RAC1 (Figure 5G) and CDC42 (Figure 5H) were unaltered. We thus conclude that in amygdala, RICH2 shows preferential GAP activity for RhoA. RICH2 deletion leads to a disinhibition of RhoA. Together with an increase in total RhoA protein, the results hint towards an increase in RhoA signaling at synaptic spines in the amygdala of RICH2 KO mice.

### Altered Spine Morphology and Actin Polymerization in the Amygdala of RICH2 KO Mice

**Figure 6:**
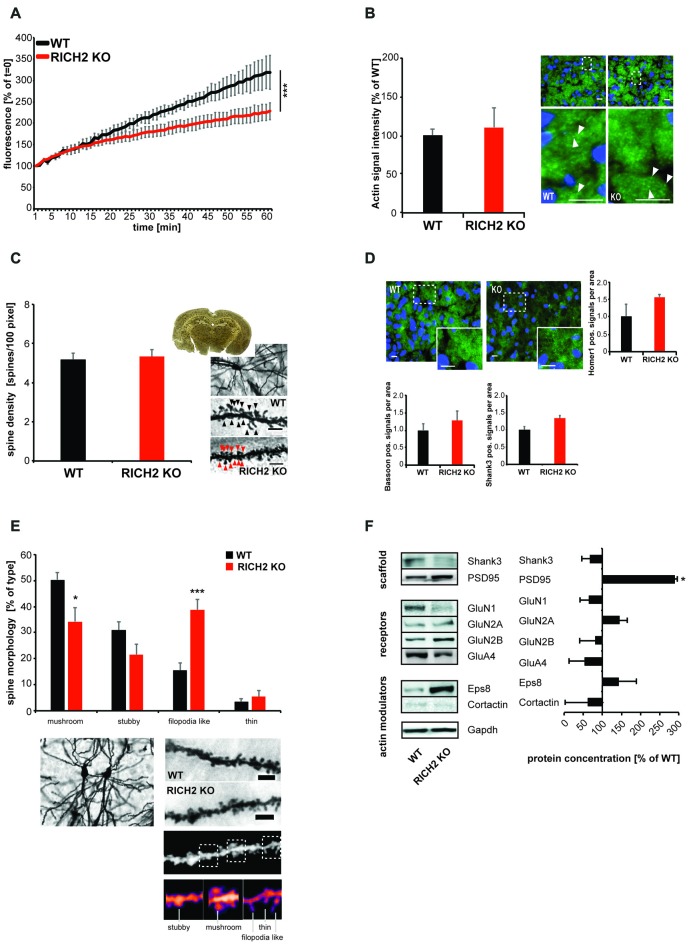
Altered actin polymerization and spine morphology in amygdala of RICH2 KO mice. **(A)** Amygdala P2 lysates from WT and RICH2 KO animals (*n* = 3) were used in an actin polymerization assay. The lysate was added to a solution with pyrene-conjugated actin and the increase in fluorescence intensity that occurs when pyrene G-actin (monomer) forms pyrene F-actin measured over a time-course of 60 min. P2 lysate from amygdala of RICH2 KO mice induces actin polymerization to a significantly lower amount compared to lysate from WT mice (ANOVA, *F*_(1,14)_ = 537.128, *p* < 0.0001). **(B)** The amount of total actin at synapses, measured by Immunohistochemistry (IHC; arrows) in the amygdala region of mice, is not significantly different between RICH2 KO and WT mice. Scale bar = 30 μm. **(C)** Spine density assessed by Golgi staining in the amygdala of mice (*n* = 10), was not significantly different in RICH2 KO mice compared to WT (scale bar = 5 μm). **(D)** Spine density assessed by quantification of HOMER1, SH3 and multiple ankyrin repeat domains 3 (SHANK3; postsynaptic markers) and BASSOON (presynaptic marker) positive signals per area was not significantly different in RICH2 KO mice compared to WT. Exemplary images show DAPI staining of nuceli (blue) and HOMER1 (green; scale bar = 25 μm). **(E)** Spine morphology assessed by Golgi staining in the amygdala of mice reveals a significant loss of mushroom shaped spines in RICH2 KO mice compared to WT and a significant increase in filopodia-like spines (Unpaired *t*-test: *p* = 0.0255 for mushroom, *p* = 0.1023 for stubby, *p* = 0.0007 for filopodia, and *p* = 0.4891 for thin (*n* = 6)). Lower panels show exemplary images and image processing for analysis of spine morphology including exemplary spines for each category. Scale bar = 5 μm. **(F)** The protein composition of spines in amygdala showed alterations in RICH2 KO mice. A significant increase in PSD95 was detected along with an increase in GluN2A (seen as trend; Unpaired *t*-test, *p* = 0.0167 for PSD95 and *p* = 0.7817 for GluN2A, *n* = 3).

Given that small GTPase signaling is involved in remodeling of the actin cytoskeleton in dendritic spines and that altered spine morphology has been reported in other brain regions in RICH2 KO mice (Sarowar et al., 2016), we next assessed, whether actin polymerization is also affected at synapses in the amygdala of RICH2 KO mice (Figures 6A,B). Amygdala P2 lysates from WT and RICH2 KO animals were used in an ELISA based actin polymerization assay. The results show that lysate from amygdala of RICH2 KO mice induces actin polymerization to a significantly lower amount compared to lysate from WT mice (Figure 6A). The fluorescent intensity of actin signals at synapses in the amygdala, however, were not significantly less in RICH2 KO mice (Figure 6B). Thus, the total levels of actin were not altered in contrast to the polymerization rate.

Given that disinhibition of RhoA is associated with repressed maintenance and elongation of spines (Tashiro et al., 2000), we next evaluated synapse density and morphology. To that end, we performed Golgi staining of brain sections from WT and RICH2 KO mice. As in other brain regions before Sarowar et al. (2016), we could not detect differences in spine density in RICH2 KO compared to WT mice (Figure 6C). This is confirmed by experiments using IHC, where we could not detect significant differences in the density of immunoreactive puncta for postsynaptic marker proteins such as HOMER1 and SHANK3 and the presynaptic marker BASSOON (Figure 6D). The morphology of spines, in contrast, is significantly altered. The number of mature (represented by the categories mushroom and stubby spines) synapses is decreased in RICH2 KO mice, while the number of immature (represented by the categories filopodia-like and thin) spines is increased. The decrease in mature spine was due to the decrease in mushroom shaped spines, without alteration in stubby shaped spines. The increase in the immature spines results from an increase in filopodia-like spines, without alteration in thin shaped spines (Figure 6E).

Along with morphological differences, the protein composition of spines in amygdala shows alterations in RICH2 KO compared to WT mice (Figure 6F). Using Western blot experiments, we detected a significant increase in PSD95. It was shown that most filopodia–spine structures contain PSD95 clusters even though these spines have thin spine heads (Okabe et al., 2001; Fan et al., 2011) and PSD95 is additionally localized to the dendritic shaft. The increase in PSD95 may thus not be conflicting with a loss of mature spines.

**Figure 7:**
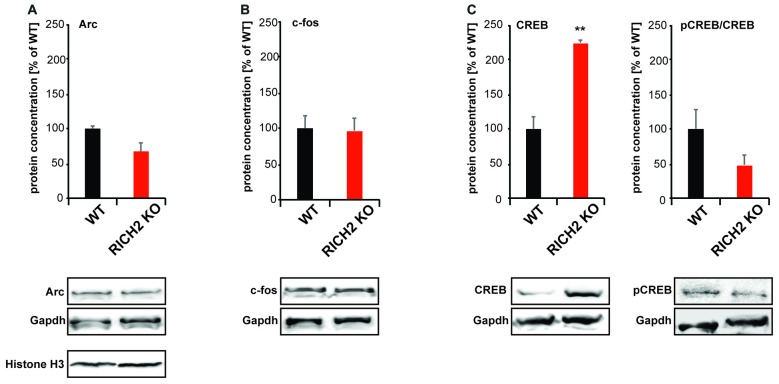
Immediate early gene (IEG) expression in RICH2 KO mice. IEG expression was analyzed in nuclear protein lysate from amygdala and normalized to GAPDH levels. No significant difference was detected in the expression of **(A)** activity-regulated cytoskeleton-associated protein (ARC; Unpaired *t*-test, *n* = 3, *p* = 0.077), and **(B)** c-FOS (Unpaired *t*-test, *n* = 3, *p* = 0.8784). A significant difference was detected in the expression of **(C)** cyclic AMP response element-binding protein (CREB; Unpaired *t*-test, *n* = 3, *p* = 0.002). The amount of phosphorylated CREB (pCREB) was unchanged (Unpaired *t*-test, *n* = 3, *p* = 0.1822). Exemplary Western blot bands are shown. Histone H3 was used a control for the presence of nuclear proteins in the lysate.

Increased activity of the amygdala, as shown before, may be expected to lead to homeostatic changes in synapse numbers and synaptic strength. However, in the amygdala of RICH2 KO mice, a shift from mature to immature synapses, no change in synapse density, and decreased synaptic actin polymerization was observed. Thus, to better understand the observed phenotype, we quantified the expression of immediate early genes in the amygdala of RICH2 KO mice under baseline conditions. Given that an increased number of c-fos positive neurons had been shown in response to novel objects before, it might be possible that activity of the amygdala is increased in general in RICH2 KO mice. Therefore, we measured IEG protein levels under baseline conditions but were unable to detect a significant difference in the expression of c-FOS and activity-regulated cytoskeleton-associated protein; (ARC; Figures 7A,B). A significant difference was detected in the expression of cyclic AMP response element-binding protein (CREB; Figure 7C). Therefore, we closer investigated the activation status of CREB. Phosphorylation activates CREB and pCREB promotes transcription in cells by recruitment of the co-activator CREB-binding protein (CBP). The amount of phosphorylated CREB (pCREB) was slightly reduced in RICH2 KO mice and thus the ratio of pCREB/CREB not significantly different between RICH2 KO and WT mice (Figure 7C).

Thus, we conclude that amygdala activity of RICH2 KO mice is only increased in response to objects, and loss of RICH2 may impair the translation of synaptic activity into lasting synaptic alterations in the amygdala of mice.

## Discussion

While anxiety is a mental state elicited in anticipation of threat, phobia is a persistent, abnormal, and irrational fear of a specific thing or situation. The ability to regulate fear responses to initially threatening cues once the value of such cues changes is critical to emotional health (Cisler et al., 2010). This regulation can be achieved through a broad range of processes, such as learning that a stimulus does not pose a threat, and is key to survival. The amygdala plays an active role in this modulation of fear responses.

Our present work focuses on further characterizing the RICH2 KO mouse that has been reported to display neophobia (Sarowar et al., 2016). RICH2 is a protein with RhoGAP activity. The Rho GTPases are a large sub group of small GTP binding proteins. RhoA, RAC1 and CDC42 are members of this family. The activity of these Rho GTPases is tightly regulated via the actions of several GAPs, GEF (guanine nucleotide exchange factors) and GDI (GDP dissociation inhibitor) proteins (Koh, 2006/2007; Tcherkezian and Lamarche-Vane, 2007). In an *in vitro* study, it was shown that RICH2 is able to promote GTP hydrolysis and thus is able to theoretically inactivate RhoA, RAC1 and CDC42 (Raynaud et al., 2014). However, *in vivo*, deletion of RICH2 has been associated with disinhibition of RAC1 in hippocampus and cerebellum of mice (Sarowar et al., 2016), which was accompanied by spine enlargement, which is in line with the reported role of RAC1 in actin dynamics within dendritic spines (Nakayama et al., 2000; Tashiro and Yuste, 2004; Sarowar and Grabrucker, 2016). RAC1 and CDC42 promote dendritic arbor growth, spine formation and maintenance whereas RhoA inhibits such activity (Newey et al., 2005).

RICH2 is also expressed in the post-synaptic compartment in amygdala neurons. Here, focusing on amygdala, we have detected over-activation of RhoA in RICH2 KO mice. In line with this, RICH2 KO mouse display less mature spines in the amygdala along with an increase in thinner, immature spines.

However, we observed that more neurons of the amygdala were activated in RICH2 KO mice, evidenced by an increase of amygdala c-FOS staining, after exposure to novel objects. The increase in c-FOS positive neurons is correlated to an increased neuronal activity in this brain region, which might be caused by alterations in other brain regions of RICH2 KO mice, such as an increase in multi-spine synapses, increased NMDAR levels, and increased mESPC amplitude and area in hippocampal neurons of RICH2 KO mice (Sarowar et al., 2016). Further, in cerebellum, an increase in multi-spine synapses was detected. Thus, given that the RICH2 KO mouse analyzed here is not an amygdala-specific conditional KO mouse, it may be possible that due to an increase in neuronal output in brain regions projecting into the amygdala, amygdala neurons of RICH2 mice show hyper-activation. Further, the lack of RICH2 in other brain areas may contribute to the neophobia of RICH2 KO mice.

Our findings are in line with previous reports showing that over-activation of the amygdala results in increased anxiety behaviors. In rodent models of anxiety, behavioral inhibition, a state characterized by increased vigilance and heightened autonomic arousal has been observed in response to novel environments and stimuli (Archer, 1973). However, behavioral inhibition has also been reported in anxious children (Kagan et al., 1988). Especially activation of neurons in the BLA is known to elicit freezing behavior (Power and McGaugh, 2002), as seen increased in RICH2 KO mice.

In the amygdala, information can be processed by intrinsic networks but also through interactions with other brain regions to integrate sensory inputs, and ultimately generate fear response outputs. In particular, we detected increased neuronal activity in the BLA. The BLA consists of a majority of glutamatergic projection neurons, most likely expressing RICH2, and a minority of local GABAergic interneurons (McDonald, 1982).

Although other parallel inputs and outputs exist (Sah et al., 2003), one of the main flows of information within the amygdala follows a serial path in the direction of the main internuclear projections (Pitkänen, 2000). There, the BLA serves as the major sensory interface, receiving sensory information from the thalamus and cortex. The medial subdivision of the CEm serves as the principal output station. Thus over-activation of the BLA may result from increased activity in upstream brain regions.

On behavioral level, we could confirm the phenotype observed previously (Sarowar et al., 2016), where we could not detect elevated anxiety in general using an Elevated Plus Maze, or signs of decreased exploratory behavior. Exploratory behavior measured by the number of rearings was also comparable to WT mice. Additionally, using a Porsolt forced swim test, we have shown before that the RICH2 KO mice do not display decreased motivational levels (Sarowar et al., 2016). Thus based on previous data and the results reported here, we conclude that RICH2 mice show a specific aversion to novel objects. The fear was not present, when novel odors were presented to RICH2 KO mouse. Further, we have not seen significant differences dependent on the shape of the object. Both, inorganic highly symmetrical shapes and also organic shapes (horse) elicited the same response. Upon presentation of a novel object, both the novel object and the already known object were avoided. This indicates that RICH2 KO mice do not habituate to objects presented in the open field during the duration of the experiments. This is in line with findings that taste neophobia evoked more c-FOS immunoreactivity than a familiar taste in the BLA (Lin et al., 2012). However, in WT mice, once the taste becomes familiar, c-FOS signals normally decrease.

Thus, one reason for the development of neophobia in RICH2 mice may be a lack of synaptic plasticity necessary for learning to distinguish between threatening and neutral objects and the habituation towards novel stimuli. Due to RICH2 regulating RhoA signaling in the amygdala, the increased neuronal activation may not be translated into proper synaptic changes in the amygdala of RICH2 KO mice. Although more neurons are activated, the amount or activation status of the IEG ARC, c-FOS and CREB present in the amygdala of RICH2 is not increased accordingly, and thus, the average level/activity per active neuron may even be reduced. Indeed, it has been shown before that RhoA, RAC1 and CDC42 are implicated in regulating transcriptional activation by Serum Response Factors (SRF) in fibroblasts (Hill et al., 1995). In neurons, Megakaryoblasic Acute Leukemia-1 (MAL/MKL1) is regulated downstream of the RhoA signaling pathway. G-actin depletion releases MAL to translocate to the nucleus (Tabuchi et al., 2005), where MAL binds SRF. *Srf* conditional brain specific knockout mice showed defects in spine morphology (Stritt and Knöll, 2010). Although it is unclear whether RhoA-SRF signaling may play a role in activity-dependent circuit rewiring in the amygdala glutamatergic neurons in association with plasticity, our results hint towards this possibility. Therefore, hyper activation of RhoA and the resulting inability to perform actin based synapse modifications, may ultimately prevent habituation to novel stimuli such as objects.

The specificity of the fear for objects may result from increased activation of neurons in brain regions delivering input coding for specific sensory modalities into the amygdala. This in turn implies that in WT mice under physiological conditions, the brain region specific activity of RICH2 for GTPases of the Rho family may provide a mechanism of crosstalk balancing activity between circuits connecting different brain regions.

## Author Contributions

TS carried out the analysis of RICH2 mice, and revised the manuscript. SG supported the behavioral analysis of mice and revised the manuscript. TMB participated in the design of the study, and contributed antibodies and reagents. AMG conceived of the study, participated in its design, coordination and data analysis, and drafted the manuscript. All authors read and approved the final manuscript.

## Conflict of Interest Statement

The authors declare that the research was conducted in the absence of any commercial or financial relationships that could be construed as a potential conflict of interest.
